# The Burnaby treatment center for mental health and addiction, a novel integrated treatment program for patients with addiction and concurrent disorders: results from a program evaluation

**DOI:** 10.1186/1472-6963-13-288

**Published:** 2013-07-30

**Authors:** Christian Schütz, Isabelle Aubé Linden, Iris Torchalla, Kathy Li, Majid Al-Desouki, Michael Krausz

**Affiliations:** 1 Centre for Health Evaluation & Outcome Sciences, Providence Health Care St. Pauls Hospital, 588-1081 Burrard St., Vancouver, BC V6Z 1Y6, Canada; 2 Department of Psychiatry, University of British Columbia, 430-5950 University Boulevard, David Strangway Building, Vancouver, BC V6T-1Z3, Canada; 3 Vancouver Coastal Health, 601 West Broadway, Vancouver, BC V5Z 4C2, Canada; 4 School of Population and Public Health, University of British Columbia, 5804 Fairview Avenue, James Mather Building, Vancouver, BC V6T 1Z3, Canada

**Keywords:** Concurrent disorders, Integrated treatment, Marginalized populations

## Abstract

**Background:**

Patients with addictions and concurrent disorders constitute the most underserved population in the system of care. There are numerous reasons why this population has so much difficulty accessing services, including behavioural issues, criminal engagement, and non-compliance with outpatient services. To improve services to this population which is marked by multiple morbidities, high mortality and insufficient access to health care, the government of British Columbia, Canada developed a program for people with both substance use disorder and one or more mental disorders who have not benefited from previous therapies.

**Method:**

In July 2008, the Burnaby Treatment Centre for Mental Health and Addiction (BCMHA), a specialized and integrated tertiary care facility, was opened. The current article provides a description of the treatment program and a clinical profile of the population.

**Results:**

The target population is being served, at intake clients present with high rates of psychopathology, childhood and adult trauma, and substance use.

**Conclusion:**

While preliminary, these results indicate, that the novel approach of the Burnaby Centre may constitute a new path towards providing effective recovery for this population.

## Background

Individuals with concurrent mental and substance use disorders tend to present with multiple physical health problems and substantial social and behavioural problems [1]. Individuals with concurrent disorders (CD) are overrepresented in forensic settings, regularly inhabit substandard housing [2,3] and constitute a significant percentage of the homeless population [4,5]. Individuals suffering from CD typically have difficulty engaging with traditional health care services and tend to rely heavily upon emergency care as their access point to the health care system [6]. The CD population exhibits extremely poor health outcomes and has a life expectancy that is considerably lower than the general population [7,8]. These and other concerns were recently emphasized by a group of leading American psychiatrists in a recent ‘call for action’ [9].

In the Canadian province of British Columbia (BC), the highest numbers of patients with CD and those with the most severe problems are found within inner-city neighbourhoods. In Vancouver, the area known as the Downtown Eastside (DTES) has a particularly high concentration of CD clients and has been the focus of considerable efforts to develop special treatment programs, including low threshold or harm reduction approaches [10]. Although there are existing treatment programs for substance abuse, mental health, and physical health issues, many health care providers in the DTES have expressed concern that these services are inaccessible to CD clients [11,12]. In longitudinal studies, having CD was associated with lower motivational readiness to change, lack of treatment engagement and attendance, and poor medication compliance [13,14]. Many of these individuals have ‘behavioural issues’, such as high impulsivity, aggression, and involvement in criminal activity [15]. These dysfunctional behaviours may be an expression of street entrenched life, mental disorders, substance intoxication/dependence, or a combination of all of these dimensions. Unfortunately, these types of behaviours will often disqualify CD clients from health services, and bring individuals into frequent contact with the criminal justice system [16]. Resources could be more effectively allocated if these concurrent conditions were treated consistently and if the available therapies were better tailored to the realities of this vulnerable population [17].

### Development of a treatment model for individuals with CD in British Columbia

Despite an influx of resources into this vulnerable neighbourhood over the past 20 years, the health concerns facing DTES residents and clients with CD throughout the province of BC have not been resolved. Therefore, in 2008, an overall consensus for significant change drove the creation of a new approach to managing the health issues of CD clients. The development of a specialized program for clients with CD was mandated by the provincial government of BC. In April 2008, funding for the development of a specialized, 100 bed provincial treatment facility was announced, and in July of 2008, the Burnaby Treatment Centre for Mental Health and Addiction (BCMHA) was opened. The founding principles of BCMHA were developed by a panel of experts with ranging specialties from substance abuse treatment, psychological therapy and rehabilitation as well as representatives from acute care, community care, and forensic services. The model of care was designed to incorporate principles of strength-based care and the concepts of assertive treatment, motivation-based treatment, time-unlimited treatment, comprehensive programming, treatment approaches tailored to the receptiveness of clients (e.g. starting at low intensity), harm reduction leading to abstinence*,* stepped care, and cultural competence and sensitivity [18]. The BCMHA emphasizes two key strategies: 1) the management of relapse and crisis as the basis of achieving recovery for patients, and 2) long-term rehabilitation-focused care, reflecting a core belief that while recovery is a long process-it is the only alternative to reduce the serious mortality in this population.

The BCMHA was deemed to be a tertiary care program and given the mandate to provide comprehensive care to individuals with CD who present with severe mental health, physical health, substance use, and behavioural issues. Comprehensive care was defined as including all stages of treatment for each dimension of care, including withdrawal management, psychiatric care (excluding emergency care), psychosocial care, and medical care (excluding emergency/acute care).

The treatment program is designed for clients to stay up to 9 months at the inpatient facility/treatment program; thus reflecting the extensive change required during recovery from concurrent disorders. Although clients are encouraged to stay 9 months, there is considerable flexibility, as some clients will require shorter involvement while others will benefit from long-term care, therefore the center does not have strict and arbitrary time limits. The treatment team consists of care providers including psychiatrists, psychologists, physicians, nurses, counsellors, health care workers, social workers, in addition to occupational therapists, art and music therapists, and providers of alternative medicine. Treatment goals are determined in team meetings with the client. Treatment is based on best evidence as provided by international treatment guidelines and reviews of treatment efficacy. Treatment includes individual and group interventions targeting specific issues such as relapse prevention, contingency management, anger management, and motivational interviewing. Interventions are offered at different levels of complexities, allowing an individual to progress from simpler, low intensity approaches to more demanding and intensive interventions. Table 1 describes the different treatment components available to clients at BCMHA. Concurrently, clients receive medication treatment for mental health and medical issues. Clients are encouraged to progress from tightly supervised medical treatment to a weekly handout of medication.

**Table 1:** BCMHA recovery and clinical pathway model

**Treatment**	**Goals**	**Treatment elements**
Recovery 1 (20–40 days)	• Complete the withdrawal management process	• Medication treatment by psychiatrists and GPs. One-to-one sessions with psychiatrists and counsellors.
	• Stabilize all medical and psychiatric disorders	
	• Stabilize sleep patterns	• Complimentary therapies e.g.
	• Reduce behavioural and emotional instability	• acupuncture and yoga.
		• Group programs include: Motivational Enhancement, Anger Management, Early Recovery - Substances (Matrix), Early Recovery - Mental Health, Emotional Boot Camp (introductory), Life Skills, Talking Circle.
	• Introduce a range of healthy habits	
	• Prepare residents to participate in structured educational, therapeutic, and recreational activities	
		• Recreational activities
Recovery 2 (90–180 days)	• Provide clients with a basic understanding of the nature of addiction and mental health problems	• Continued medical follow-up, therapy sessions and complimentary therapies.
	• Teach clients techniques for self- managing emotions and behaviours	• Group programs include: Emotional Bootcamp, Anger Management, Seeking Safety, Cognitive Behavioural Therapy (for psychosis and affective disorders), Emotional Boot Camp, Mindfulness, Relapse Prevention (Matrix), Living Free, Life Skills, Talking Circle, Stages of Change,
	• Allow clients to explore a range of creative and recreational activities	
	• Introduce clients to techniques for managing substance use and mental health problems	
	• Work on developing a personal strengths inventory	
		• Hep C treatment group.
		• Recreational activities and Art therapy
Recovery 3 (40–60 days)	• Identify a secure housing situation	• Continuation of Recovery 2 programs as well as Life Management, Stepping Up and Stepping Out.
	• Establish a financial and vocational plan	
	• Connecting the client with community organizations and resources, including connecting clients with the Ministry of Housing and Income Assistance (former MIEA) and other providers	
		• Community activity and involvement is supported.
		• Self-medication plans initiated.
	• Developing and implementing a relapse prevention plan, including connections with treatment providers in the community as appropriate	

The Provincial Health Services Authority (PHSA), who established access protocols under which the five regional health authorities in British Columbia could refer CD clients to the BCMHA, organizes the referral process for the BCMHA. According to the access protocol, the patients must have failed other programs on a regional level and must have significant issues in each of the four identified domains: mental health, substance use, physical health and behavioural. Furthermore, clients eligible for admission must have been unable to adequately engage with, receive services from, or benefit from traditional mental health and addiction programs.

The centre’s mandate was to meet the needs of the vulnerable population in BC, and to help a population whose complexity of daily living made it difficult for them to benefit from existing services. As clients at the BCMHA are both difficult to engage in treatment and present with extremely challenging combinations of health problems, the present study’s objective is to describe the needs of these patients by presenting baseline (intake) data to outline the level of mental illness in this population and to inform planning and tailoring of treatment services for these difficult to treat clients. A description of the characteristics of the client population and their initial responses to the intervention are presented.

## Methods

This program evaluation consisted of a baseline assessment, and a follow up assessment at 6 months. Baseline data were collected from June 2009 to January 2010, and follow up assessment began in December 2009 and were completed in March 2010. All potential participants in the study were adult residents of the BCMHA who had been admitted in accordance with a standardized access process that was regulated by the BC Provincial Health Services Authority [19]. One hundred and twenty-eight clients who were consecutively admitted to the BCMHA were contacted to take part in an assessment, and assessed for eligibility by the intake team. We completed the baseline assessment for the pre-test within 6 weeks of intake. Clients were asked to respond in their baseline assessment regarding their status at intake. A total of 112 clients consented to participate in the study and 92 participants completed the minimal baseline assessment. Baseline information included information on mental disorders, substance use patterns, and health status. Due to funding restrictions that prevented tracking of patients who were discharged or had dropped out of the study, follow-up interviews were completed only of individuals who were still at the treatment centre. This study was reviewed and approved by the University of British Columbia Research Ethics Board.

We collected demographic information, which included age, gender, ethnicity, education, recent employment, and housing situation.

Mini-International Neuropsychiatric Interview (MINI) Plus [20] is a structured clinical interview to assess current and lifetime substance use and mental disorders according to the criteria of the Diagnostic and Statistical Manual, 4^th^ edition (DSM-IV).

Childhood Trauma Questionnaire, short form (CTQ-SF) [21] is a retrospective self-report inventory that assessed different types of childhood maltreatment on five subscales: Physical Abuse, Emotional Abuse, Sexual Abuse, Physical Neglect, and Emotional Neglect. The questionnaire consists of 28 items answered on a 5-point Likert scale, including three items to assess minimization/denial. We adopted the severity classification proposed by the developers.

Trauma History Questionnaire (THQ) [22] is a 24-item self-report measure that examines experiences with potentially traumatic events, including crime-related events (e.g., robbery, burglary), general disasters (e.g., accidents, natural or man-made disasters, war, injury, life-threatening illnesses, or deaths of others), and sexual and physical assault. For each item, the clients were asked to indicate the frequency and at what age they had experienced the event.

The Brief Symptom Inventory (BSI) [23] is a 53 item self-report questionnaire that measures nine dimensions of psychological distress over the past 7 days using a five-point Likert scale. The nine dimensions are: Somatization, Obsession-Compulsion, Interpersonal Sensitivity, Depression, Anxiety, Hostility, Phobic Anxiety, Paranoid Ideation and Psychoticism. In addition to the average score for each individual dimension, we calculated the Global Severity Index (the average score of all items combined) as a measure of overall current distress.

The Maudsley Addiction Profile (MAP) [24] is a self-report measurement that assesses current substance use related problem behaviours. Participants were asked to indicate the frequency, amount, and route of administration of alcohol, cocaine (powder or crack cocaine), cannabis, opioids (heroin, nonprescribed methadone, or nonprescribed opioids), amphetamines (amphetamines or crystal methamphetamines), and nonprescribed benzodiazepines they used in the past 30 days.

Descriptive analyses were used to describe the sample, including numbers and percentages for dichotomized sociodemographic and clinical variables, and means and standard deviations (SD) for continuous variables. Comparison between baseline and follow-up MAP and BSI measures were assessed as indicators of the client’s progress in treatment. Within this matched analysis, paired t-test and chi-square test were employed to examine the mean differences and the differences in proportions. All reported p-values are two-tailed and significance was set at p ≤ 05. Analysis was performed using SAS version 9.1 (SAS Institute, Inc, Cary, North Carolina).

## Results

Ninety-two participants completed the baseline assessment. The mean age at baseline was 40.2 years, and 21.7% identified as Aboriginal. Complete client demographic characteristics can be found in Table 2. The average length of stay at BCHMA for all clients discharged in 2010 was 4.8 months.

**Table 2:** Client’s demographic characteristics

**Variables**	**N = 92 (%)**
Mean Age (SD)	40.2 (10.3)
(Range)	(21–63)
Gender	
Male	60 (65.2%)
Female	32 (34.8%)
Ethnicity	
White	64 (69.6%)
Aboriginal	20 (21.7%)
Other	8 (8.7%)
Education	
≥ high school exam	27 (29.3%)
< high school exam	65 (70.7%)
Recent Employment	
No	81 (88.0%)
Yes	11 (12.0%)
Housing Status	
Fixed Address	29 (31.5%)
SRO*	12 (13.1%)
Shelters/Surfing	22 (23.9%)
Living on the street/homeless	29 (31.5%)

*SRO: Single Room Occupancy (generally substandard housing).

On the BSI, clients at intake scored highest in dimensions of obsession-compulsion (2.11) and depression (2.08). However, symptoms for all dimensions on the BSI were high. Full scores for the BSI can be found in Table 3.

**Table 3:** BSI dimensions scores and composite GSI score, and comparison of baseline and follow-up (FU) data for n = 47

**BSI Dimension**	**Baseline, all clients (N = 92)**	**Baseline of those assessed at FU (n = 47)**	**Follow up (n = 47)**	**p**
	**Mean (SD)**	**Mean (SD)**	**Mean (SD)**	
Somatization	1.43 (1.02)	1.69 (1.04)	1.14 (0.84)	.006
Obsess.-Compuls.	2.11 (1.05)	2.29 (1.11)	1.67 (1.09)	.0004
Interp. Sensitivity	1.92 (1.20)	2.07 (1.22)	1.50 (1.14)	.0014
Depression	2.08 (1.16)	2.21 (1.17)	1.40 (1.06)	<.0001
Anxiety	1.90 (1.13)	2.11 (1.20)	1.40 (1.08)	<.0001
Hostility	1.48 (1.05)	1.59 (1.09)	1.19 (1.02)	.0258
Phobic Anxiety	1.44 (1.15)	1.66 (1.24)	0.97 (0.99)	<.0001
Paranoid Ideation	1.77 (1.06)	1.96 (1.10)	1.52 (0.94)	.0024
Psychoticism	1.77 (0.96)	1.83 (0.99)	1.29 (0.99)	.0004
GSI	1.78 (0.91)	1.94 (0.95)	1.31 (0.83)	<.0001

Extremely high rates of trauma were found in this population using the CTQ and the THQ. More than half of the sample had experiences at least one form of trauma in their childhood; the most frequently reported experience was emotional abuse. On the THQ, general disasters and crime related events were most frequently reported. The full results for trauma histories are presented in Table 4.

**Table 4:** Results of the Childhood Trauma Questionnaire (CTQ, n = 75) and the Trauma History Questionnaire (THQ, n = 84)

**CTQ subscales**	**n (%)**
**Emotional Abuse**	
no to low	24 (32.0%)
moderate to severe	51 (68.0%)
**Physical Abuse**	
no to low	26 (34.7%)
moderate to severe	49 (65.3%)
**Sexual Abuse**	
no to low	33 (44.0%)
moderate to severe	42 (56.0%)
**Emotional Neglect**	
no to low	32 (42.7%)
moderate to severe	43 (57.3%)
**Physical Neglect**	
no to low	32 (42.7%)
moderate to severe	43 (57.3%)
**Trauma History Questionnaire**	**n (%)**
**Physical Assault** (yes)	55 (65.5%)
**Sexual Assault** (yes)	37 (44.0%)
**Crime–related events** (yes)	65 (77.4%)
**General Disaster** (yes)	69 (82.1%)

Results from the MAP revealed high rates of substance use, with crack or powder cocaine use the most common substance used at 65.2%. The complete list of substances used is found in Table 5.

**Table 5:** Prevalence of substance use at baseline for all clients, and comparisons of baseline and follow-up substance use for individuals available for follow up (n = 47)

**Substances**	**Baseline, all clients N = 92**	**Baseline of those assessed at FU n = 47 (%)**	**Follow-up n = 47 (%)**	**p**
Alcohol	45 (48.9%)	16 (34.0%)	5 (10.6%)	.006
Heroin	31 (33.7%)	15 (31.9%)	6 (12.8%)	.026
Crack or powder cocaine	60 (65.2%)	30 (63.8%)	9 (19.1%)	<.0001
Illicit methadone	5 (5.4%)	4 (8.5%)	1 (2.1%)	.168
Illicit benzodiazepines	14 (15.2%)	7 (14.9%)	3 (6.4%)	.181
Amphetamine	12 (13.0%)	7 (14.9%)	3 (6.4%)	.181
Injection Drug Use	32 (34.8%)	-	-	
Sharing syringes	2 (2.2%)	-	-	

The MINI revealed that for lifetime mental disorders, major depressive episodes was the most frequently reported diagnosis (64.8%). For substance use disorders, drug dependence (78.4%) was more frequently reported than alcohol dependence (65.9%). The complete list of lifetime prevalence rates of mental and substance use disorders can be found in Table 6.

**Table 6:** Patient’s lifetime prevalence rates of DSM-IV based mental and substance use disorder diagnoses

**Diagnoses**	**N = 88 (%)**
Major depressive episode	57 (64.8%)
Manic(Hypo-manic) episode	36 (40.9%)
Dysthymia	2 (2.3%)
Psychotic episode not induced by substances	49 (55.7%)
Panic disorder	30 (34.1%)
Agoraphobia	43 (48.9%)
Posttraumatic stress disorder	38 (43.2%)
Antisocial-personality disorder	32 (36.4%)
Alcohol dependence	58 (65.9%)
Drug dependence	69 (78.4%)
History of suicide attempt(s)	52 (59.1%)

A total of 47 clients (51%) completed the follow-up assessment after six months. There was a significant reduction in psychopathology symptoms from intake to 6 months across all BSI dimensions. The means and SDs of the baseline and the follow-up BSI scores can be found in Table 3, along with the p-values for the comparisons. Specifically, participants improved in dimensions of somatization (t(46) = 4.489, p = .006), obsessive-compulsive (t(46) = 3.900, p = .0004), interpersonal sensitivity (t(46) = 3.428, p = .0014), depression (t(46) = 5.239, p < .0001), anxiety (t(46) = 4.507, p = <.0001), hostility (t(46) = 2.304, p = .0258), phobic anxiety (t(46) = 4.778, p < .0001), paranoid ideation (t(46) = 3.209, p = .0024), psychoticism (t(46) = 3.739, p = .0004), and the GSI (t(46) = 5.204, p < .0001). Even after using a Bonferroni correction to account for multiple testing (resulting in an alpha of .005), the differences from baseline to follow-up remained significant on all dimensions except somatisation and hostility.

Results from the MAP indicated reduction of substance use to overall minimal use. The numbers and percentages of substance use at baseline versus follow-up are presented in Table 5. Specifically, the rates decreased significantly for alcohol (χ2(1) = 7.42, p = .006), heroin (χ2(1) = 4.97, p = .026), and cocaine (χ2(1) = 19.3, p < .0001). Using a Bonferroni correction resulted in an alpha of .0083, indicating that the changes remained significant for alcohol and cocaine use. The differences from baseline to follow-up were not significant for illicit methadone (χ2(1) = 1.90, p = .168), benzodiazepines (χ2(1) = 1.79, p = .181), and amphetamines (χ2(1) = 1.79, p = .181).

## Discussion

The present study focused on describing a residential treatment program designed to address the needs of individuals with chronic and severe concurrent conditions. The data from the baseline assessments clearly presents that this population was suffering from severe concurrent disorders at the time of intake to the clinic. Compared to normative data provided by the authors of the BSI, the psychopathology distress not only exceeded the psychopathology of the general population, but also the psychopathology found among psychiatric inpatients [23]. The high levels of mental illness, concurrent disorders, and multiple traumatic experiences present in this population clearly demonstrate the importance of comprehensive and integrated care to achieve sustainable recovery.

Health care systems traditionally focus on mental health and addiction separately based on different philosophies of care. While many mental health services are increasing their treatment to include individuals with “mild to moderate” forms of substance dependence, and addiction services are increasing their treatment to include individuals with mild to moderate mental disorders, it is the individual with complex, severe, and concurrent conditions, that is still caught in the gap left between two incomplete and often incompatible treatment models [1,17]. However, increasingly are integrated treatment approaches of concurrent substance use and mental disorders accepted to be the most promising and best practice strategy [25].

Reflecting on the presented health issues in this sample, it is important to note the severity of problems present, and yet the limited access to care. This sample displayed major mental illness, trauma, and substance use, and although each of these issues requires medical attention, the clients’ access to care prior to involvement with BCMHA was extremely limited. The high level of traumatic experiences from early childhood to adulthood presents a chronic condition that needs more attention both as a contributing factor to mental disorder and substance use order and as a potential roadblock to accessing services. Integrated treatment approaches that address both trauma/PTSD and substance use have shown some initial promising results but need to be further refined and evaluated [26]. Recovery and reintegration into society is only possible with a comprehensive and integrated long-term concept, including housing and social support. Stimulant use in this population is high as they are low in cost and broadly available, resulting in chronic substance use patterns that include a range of psychotropic substances and routine polysubstance use. Therefore, treatment must address polysubstance use, rather than dependence to one substance in particular.

As a result of the referral process, it is expected that these clients represent the most complex populations in mental health and addiction care in BC. BC’s population is concentrated with about 60% in the metropolitan area of Vancouver and the lower mainland. It seems that access is more limited from some areas, such as very rural areas compared to cities and the metropolitan area of Vancouver. This was not specifically assessed, but may constitute an area of interest for further follow up.

The manner in which the BCMHA program understands and responds to relapse is central to the program. Relapse is a regular occurrence in substance use and CD clients, and was often the reason that BCMHA clients had been discharged from other programs or from housing facilities. Discharge often resulted in these clients living on the street despite their severe mental, addictive, and physical illnesses. From our experience with BCMHA we have learned that a comprehensive program can be used to achieve significant gains, as shown by the improvement in psychopathological symptoms and decreased substance use even before clients achieved abstinence or before mental health problems are fully resolved. These data suggest that it is possible to provide effective integrated care for patients who have not achieved full abstinence and who require longer-term care before being able to stay abstinent, as demonstrated by an average length of stay of 4.8 months, as compared to many 12-week programs.

### Limitations

Our study has some methodological limitations that warrant discussion. Two important domains were not addressed sufficiently given the major health concerns in this population. First, the level of cognitive functioning and all related conditions, due to mild traumatic brain injury, and fetal alcohol spectrum disorder, etc. Second, the presence of possible Axis II personality and developmental disorders. The assessment of both domains is time consuming and needs highly trained interviewers. These areas need to be the focus of future studies. The high rates of chronic substance use behaviours and disorders raise concerns about the interference of substance use symptoms (e.g., intoxication or withdrawal) with proper psychiatric assessment. Although the reported mental disorder symptoms and diagnoses are based on standardized assessments, over- or underestimation cannot be excluded, given the level of our patient’s impairment and the complexity of concurrent conditions. Furthermore, participant’s information on both baseline and follow-up substance use behaviours was derived via self-reports without any biochemical validation, and thus may be affected by reporting bias. Similarly, self-reported information on trauma histories in childhood and adulthood was not confirmed by external sources and may be over- or underestimated in our study.

A major limitation of the follow-up results is the fact that they were based on the minority of patients who were still present in the facility at six months. Not only does this attrition result in low statistical power, but it is also very likely that these individuals are not representative of all patients accessing the BCMHA. As such, our follow-up results need to be regarded as preliminary and suggestive, and have to be confirmed with more systematic data collection that assesses mental health and substance use outcomes over a longer follow-up time period using an intention-to-treat approach. Finally, lack of a control condition, treatment attendance and compliance measures for patients, and treatment fidelity or manual adherence measures for staff limits conclusions regarding the actual impact of the specific psychiatric intervention. However, we hope that this initial data will provide incentive for a more comprehensive analysis of the situation of individuals with complex concurrent disorders.

## Conclusions

As indicated earlier, the eligibility criteria for the BCMHA includes demonstrated failure in other treatment programs; BCMHA serves as a “last resort” tertiary care facility. There are no comparable, specialized programs in Canada focussing on these high need clients [27], which makes the BCMHA particularly interesting and challenging from both a system and a research perspective. With an interdisciplinary approach, it is possible to retain and support clients with the highest complexity of mental and substance disorders into treatment and achieve significant improvements. The current study does not seek to identify the roadblocks to accessing care, however, this is an important feature that needs to be further investigated into, as appropriate health care delivery is only achieved if and once appropriate services are accessed.

Many of the patients who participated in this study were never appropriately assessed before admission to BCMHA. Although a high prevalence of traumatic experiences or impairments in the cognitive functioning were known about these clients, no neuropsychological tests, brain imaging, or standardized psychometric tests in those fields were documented in the files or mentioned by them. Without standardized assessment or systematic outcome control it is hard to develop an appropriate care plan and provide the necessary supports. The need for better multi-dimensional assessment is a core prerequisite of any professional care for this population in the future. The consequences of all these poor health outcomes are devastating for this high need and high risk population, their families, their peers, and especially their children. The lack of appropriate capacity and quality of care needs to be addressed as a health crisis. For individuals with the highest morbidity, access and quality of care need to be improved. The approach offered by the BCMHA may constitute a decisive step towards this direction.

## Competing interests

The authors declare that they have no competing interests.

## Authors’ contributions

CS: Co-PI, conceptualizing the study, writing protocol, supervising analysis, conceptualizing and writing the manuscript, and editing. IL: organizing the assessments, conducting literature review, interpretation and discussion of results, writing and editing of the manuscript. IT: supervising data management, training interviewer, conducting literature review, interpretation and discussion of results, writing and editing of the manuscript. KL: data management, statistical analysis of the data. MA: developed Table 1, and editing of the manuscript. MK: PI of the evaluation study, writing protocol, editing the manuscript. All authors read and approved the final manuscript.

## Pre-publication history

The pre-publication history for this paper can be accessed here:

http://www.biomedcentral.com/1472-6963/13/288/prepub
